# Comparisons Between Normobaric Normoxic and Hypoxic Recovery on Post-exercise Hemodynamics After Sprint Interval Cycling in Hypoxia

**DOI:** 10.3389/fphys.2022.843574

**Published:** 2022-03-24

**Authors:** Masahiro Horiuchi, Ayano Nishida, Shohei Dobashi, Katsuhiro Koyama

**Affiliations:** ^1^ Division of Human Environmental Science, Mount Fuji Research Institute, Fuji-yoshida, Japan; ^2^ Graduate School of Education, University of Yamanashi, Kofu, Japan; ^3^ Graduate School of Health and Sports Science, Juntendo University, Inzai, Japan; ^4^ Gradulate School Department of Interdisciplinary Research, University of Yamanashi, Kofu, Japan; ^5^ Faculty of Sport Science, Yamanashi Gakuin University, Kofu, Japan

**Keywords:** blood lactate concentration, hypotension, hypoxic vasodilation, mean arterial pressure, near infrared spectroscopy, tissue oxygen saturation

## Abstract

The aim of this study was to investigate the effects of either normoxic or hypoxic recovery condition on post-exercise hemodynamics after sprint interval leg cycling exercise rather than hemodynamics during exercise. The participants performed five sets of leg cycling with a maximal effort (30 s exercise for each set) with a 4-min recovery of unloaded cycling between the sets in hypoxia [fraction of inspired oxygen (FiO_2_) = 0.145]. The load during pedaling corresponded to 7.5% of the individual’s body weight at the first set, and it gradually reduced from 6.5 to 5.5%, 4.5, and 3.5% for the second to fifth sets. After exercise, the participants rested in a sitting position for 30 min under normoxia (room-air) or hypoxia. Mean arterial pressure decreased over time during recovery (*p* < 0.001) with no condition and interaction effects (*p* > 0.05). Compared to pre-exercise values, at 30 min after exercise, mean arterial pressure decreased by 5.6 ± 4.8 mmHg (mean ± standard deviation) during hypoxic recovery, and by 5.3 ± 4.6 mmHg during normoxic recovery. Peripheral arterial oxygen saturation (SpO_2_) at all time points (5, 10, 20, and 30 min) during hypoxic recovery was lower than during normoxic recovery (all *p* < 0.05). The area under the hyperemic curve of tissue oxygen saturation (StO_2_) at vastus lateralis defined as reperfusion curve above the baseline values during hypoxic recovery was lower than during normoxic recovery (*p* < 0.05). Collectively, post-exercise hypotension after sprint interval leg cycling exercise was not affected by either normoxic or hypoxic recovery despite marked differences in SpO_2_ and StO_2_ during recovery between the two conditions.

## Introduction

After an acute single bout of physical exercise irrespective of exercise mode, intensity, and duration, arterial blood pressure decreases for approximately up to 2 h compared to the baseline (pre-exercise) levels; this phenomenon is called “Post Exercise Hypotension (PEH)” (Halliwill et al., 2014). While previous studies about PEH have primarily used moderate-intensity prolonged exercise (about 60% of peak aerobic capacity for up to 60 min) (McCord et al., 2006; Francisco et al., 2021), few existing studies have investigated PEH and related phenomena using short-duration high-intensity exercise (Lacewell et al., 2014). However, after such high-intensity exercise (e.g., Wingate test, or sprint interval exercise), the incidence rate of presyncope symptoms was more than 50% among the individuals (Halliwill et al., 2014). Additionally, as orthostatic tolerance is reduced at high altitude (Rowell and Seals, 1990; Nicholas et al., 1992; Westendorp et al., 1997), it can be expected that the risk of presyncope or syncope might increase when exercise and/or recovery is performed at a high altitude.

However, only a few studies have assessed the impact of hypoxic exercise on PEH. PEH was more significant in 2-h aerobic exercise (Horiuchi et al., 2016) or high-intensity resistance exercise under hypoxia versus normoxia (Horiuchi et al., 2018) possibly due to hypoxic-induced vasodilation (Joyner and Casey, 2014). Another study found that there were no significant differences in the occurrence of PEH between hypoxic or normoxic 45-min aerobic exercises (Kleinnibbelink et al., 2020). The different exercise modes and recovery conditions (normoxia or hypoxia) make it difficult to obtain consensus (Horiuchi et al., 2016; Horiuchi et al., 2018; Kleinnibbelink et al., 2020). To the best of our knowledge, there have been no studies on PEH after a sprint interval leg cycling exercise performed in hypoxia.

Exercise-induced arterial hypoxemia has been observed both at higher altitude and sea level, but the magnitude of exercise-induced arterial hypoxemia was greater at high altitude than sea level (Chapman et al., 1999). Recovery from arterial hypoxemia in the hours after exercise may be important in some cases since arterial hypoxemia is associated with the occurrence or the severity of acute mountain sickness, which is mainly characterized by headache (Erba et al., 2004; Nespoulet et al., 2012). In contrast, when considering practical implications of exercise at high-altitude, continuous hypoxic stress (both during exercise and recovery) may be more effective for athletes to generate specific training adaptations (Puype et al., 2013; Gibala and Hawley, 2017). However, no studies have examined the effect of different recovery conditions (e.g., normoxia vs. hypoxia) on post-exercise hemodynamics after a sprint interval leg cycling exercise in hypoxia. A previous study reported that elevated post-exercise oxygen consumption was not associated with PEH, although their study was conducted under normoxia with a moderate exercise intensity (Williams et al., 2005); therefore, it is still uncertain about a relationship between PEH and post-exercise oxygen consumption (e.g., systemic and muscle oxygenation).

Accordingly, the aim of this study was to investigate the effects of either normobaric normoxic or hypoxic recovery condition on post-exercise hemodynamics, systemic and muscle oxygenation after sprint interval leg cycling exercise in hypoxia. We hypothesized that PEH would occur in both recovery conditions, but that the magnitude of PEH would be greater during hypoxic recovery.

## Materials and Methods

### Sample Size and Participants

Based on a previous study that observed decreases in mean arterial pressure (MAP) after hypoxic exercise (Horiuchi et al., 2016), a sample size estimation for the primary analysis (MAP) indicated that 10 participants were needed to produce an 80% chance of obtaining statistical significance at the level of 0.05 (G Power 3.1) (Faul et al., 2007).

Ten healthy young men with a mean age of 21 ± 1 years, a height of 174 ± 5 cm, and a body mass of 66.8 ± 8.7 kg [mean ± standard deviation (SD)] were enrolled. The participants engaged in regular physical activity (1–2 h per day, 3–5 days per week). None of the participants were exposed to an altitude higher than 1,500 m within 6 months prior to the study, and had a history of cardiovascular or orthopedic disease. They were non-smokers. After detailed explanations regarding the study, including the procedures, possible risks, and benefits of participation, a written informed consent was obtained from each participant. This study was approved by the Ethical Committee of the University of Yamanashi, in Japan, and was performed in accordance with the guidelines of the Declaration of Helsinki (No. 201509).

### Experimental Procedures

**FIGURE 1:**
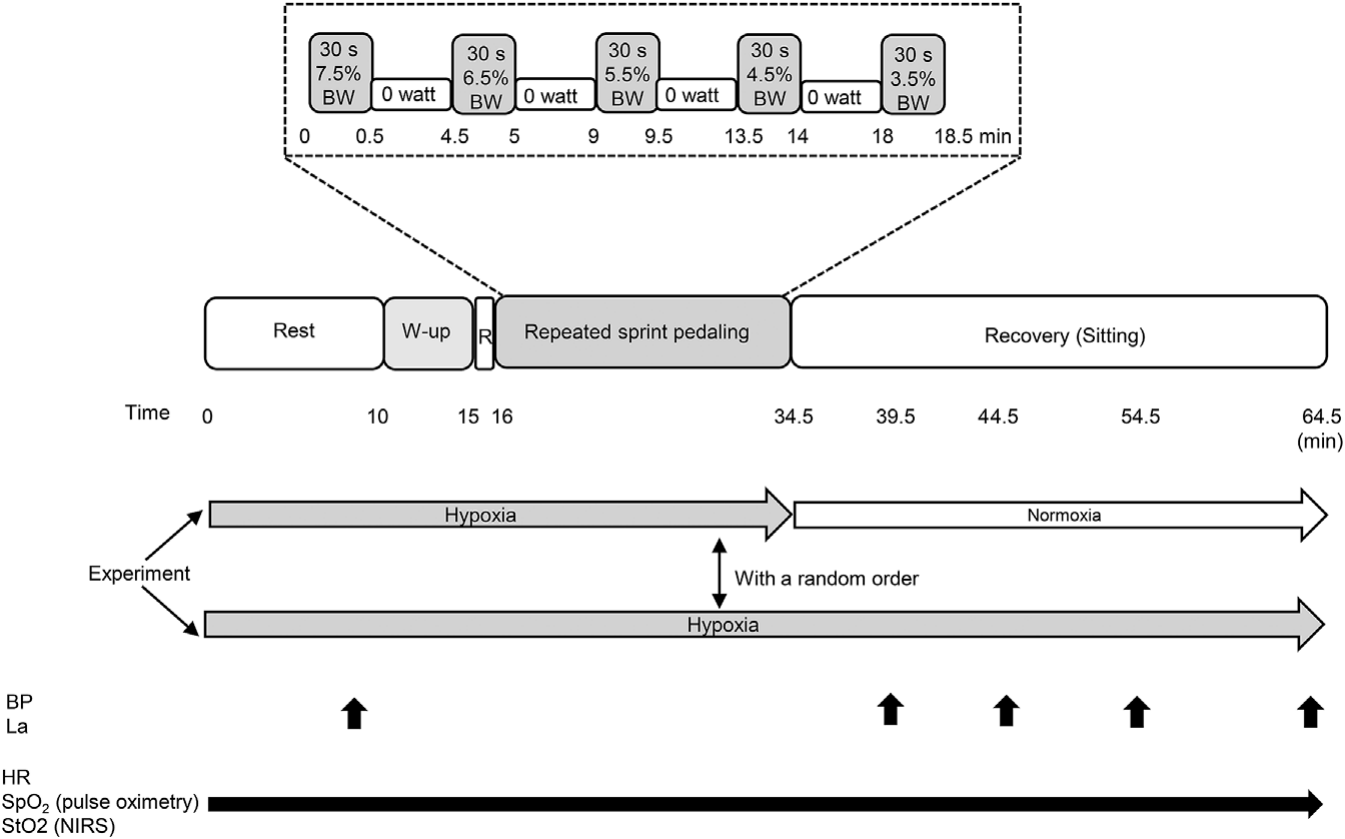
Experimental protocol of the present study. BW, body weight; W-up, warm up; R, rest; BP, blood pressure; La, blood lactate concentration; HR, heart rate; SpO_2_, arterial oxygen saturation; StO_2_, tissue oxygen saturation. Black arrows indicate the measuring points and periods.

The participants were asked to abstain from caffeinated beverages for 12 h, strenuous exercise and alcohol consumption for 24 h before each session. The room temperature was set at 24 ± 1°C, and external stimuli were minimized. All experiments were performed using a cycle ergometer (Powermax V, Combi Co. Ltd., Tokyo, Japan). On the first visit, they familiarized themselves with 1) leg cycling with maximal pedaling revolutions while wearing all the devices as well as 2) the BP measurement and blood sampling. On the second and third visits, participants performed main experiment. The experiment consisted of three consecutive protocols; 1) a 10-min sitting/resting period, 2) warm-up cycling (5-min at the pedaling load corresponded to a 2.0% of body weight at 80 revolutions per minute of pedaling frequency, including maximal pedaling for 3 s at 2 and 4 min), followed by 3) the five sets of the sprint interval leg cycling exercise in hypoxia. In the normobaric hypoxic recovery condition, participants inspired hypoxic gas vias tube attached on a face mask, from a tent, while in the normobaric normoxic recovery condition, after the five sets of the sprint interval leg cycling exercise in hypoxia, the tube was taken out from the tent immediately, then, participants could inspire normobaric normoxic gas (room air). The load during pedaling corresponded to 7.5% of the individual’s body weight at the first set, and it gradually reduced from 6.5 to 5.5%, 4.5, and 3.5% of body weight in the second to third, fourth, and fifth sets, respectively. During each five set, participants performed leg cycling for 30 s. The applied load was set according to a previous study (Kasai et al., 2015). In the preliminary test at our laboratory, we decided that decremental settings of the load was an optimal protocol to allow the participants to produce the maximal mean power despite fatigue. A 4-min recovery at 0 W (unloaded cycling at 60 revolutions per minute) was inserted between the sets. After the five sets of leg cycling exercise, they rested for 30-min sitting recovery period: in 1) normobaric normoxia (room air; equivalent to 400 m), and 2) normobaric hypoxia [fraction of inspired oxygen (FiO_2_) = 0.145; equivalent to 3,000 m]. These two tests were performed in a random order with a 1-week wash out period as shown in Figure 1.

A commercial hypoxic generator (Hypoxico Everest Summit II: Will Co., Ltd., Tokyo, Japan) supplied hypoxic gas based on a previous study (Horiuchi et al., 2017). Briefly, hypoxic gas was supplied into a custom-made tent (approximately 4,000 L) *via* one tube connected to the gas generator, and hypoxic gas was mixed in the tent. Participants inspired the hypoxic gas *via* another tube from the tent. They wore a two-way, non-rebreathing valve (for inspired and expired gas, respectively) and face mask (Horiuchi et al., 2017). Participants were blinded to inspire normoxic or hypoxic gas during recovery. The inspired oxygen concentration was verified before and after each experiment (AE-310; Minato Medical Science, Osaka, Japan).

### Measurements

The last 2 min of a 10-min resting hypoxic exposure was used to measure hypoxic baseline values before exercise. Blood pressure (BP) and blood lactate concentration (La) were measured at baseline, and at 5, 10, 20, and 30 min during recovery. BP was measured using the oscillometric method (HEM-907, OMRON, Tokyo, Japan). Resting BP was measured twice, and the average of the two measurements was considered as the participant’s BP value. We also confirmed the difference in the systolic BP or diastolic BP was <5 mmHg compared to the values of one before measurement at rest (Horiuchi et al., 2018). During the recovery period, BP was measured at 5, 10, 20, and 30 min of the period. Fingertip blood samples (0.3 μL) were taken to measure La (Lactate Pro 2LT-1730; Arkray, Tokyo, Japan). Heart rate (HR) and peripheral arterial oxygen saturation (SpO_2_) were continuously monitored throughout the experiment using a wireless HR monitor (RS 800CX, Polar Electro, Japan) and pulse oximeter (TM-256, A&D, Tokyo, Japan), respectively. The probe of pulse oximeter was attached on the left middle finger. Local tissue oxygenation profiles of the vastus lateralis muscle were also continuously measured at 1-s intervals throughout the experiment using NIRS device (BOML1TRW, Omegawave, Tokyo, Japan) (Horiuchi et al., 2017). This instrument uses three laser diodes (780, 810, and 830 nm) and calculates the relative levels of oxygenated and deoxygenated hemoglobin (Hb) in the tissue according to the modified Beer–Lambert law. The oxygen saturation of the skeletal muscle (StO_2_) was calculated by dividing oxygenated Hb by the total Hb, and was expressed as percentage (Horiuchi et al., 2017). NIRS optodes were placed on the lower third of the vastus lateralis muscle. The probe holder contained one light source probe, and two detectors were placed 2.0 cm (detector 1) and 4 cm (detector 2) away from the source at the vastus lateralis. Hb concentrations received by detector 1 were subtracted from those received by detector 2. This procedure minimized the influence of skin blood flow. Since NIRS signals can reach half the depth of the distance between the probe and detector, it can traverse at a depth of 10–20 mm at the target muscle.

### Data Analysis

The mean arterial pressure (MAP) was calculated using the following equation (Horiuchi et al., 2018):

$$\mathrm{MAP}=\frac{\left(\mathrm{systoloc BP}\left[\mathrm{SBP}\right]-\mathrm{distolic BP}\left[\mathrm{DBP}\right]\right)}{3}+\mathrm{DBP}$$

The values of HR and SpO_2_ were adopted at baseline, and at 5, 10, 20, and 30 min of recovery for further analysis. These values were averaged during the last 1 min at the sitting baseline and during the 10 s immediately after each recovery period.

Baseline values of StO_2_ were determined by the average value during the last 5 min prior to the warm-up exercise. The minimum StO_2_ values were defined as the lowest StO_2_ values during the five sets of the sprint interval leg cycling exercise. The hyperemic phase (i.e., the time from baseline values to the end of 30 min of recovery) was analyzed for the peak StO_2_ (%) and the area under the hyperemic curve (AUC; % s). The peak StO_2_ value was defined as the highest StO_2_ value achieved during the hyperemic phase. The AUC was calculated as the total area under the reperfusion curve above the baseline values (Soares et al., 2018; Horiuchi and Okita, 2020). The mean power was calculated for each set of sprint cycling, and the mean values of five sets was identified as exercise performance.

### Statistics

Data are presented mean ± standard deviation. Statistical analyses were performed using the R programming language (RKWard, Version 0.7.2). A paired *t*-test was performed to compare the sprint interval leg cycling exercise performance and NIRS indices between the two conditions. A two-way (time × recovery condition) repeated measures analysis of variance (ANOVA) was used to compare time course changes in other physiological variables (SBP, DBP, MAP, HR, La, and SpO_2_). Effect size was calculated as Cohen’s d and η^2^, defined as a trivial (<0.2), small (0.2), moderate (0.5), or large (0.8) for paired *t*-test “Cohen’s d” (Hopkins et al., 2009) and as small (*η* ^2^ = 0.01), medium (*η* ^2^ = 0.06), and large (*η* ^2^ = 0.14) for ANOVA “*η* ^2^” effects (Lakens, 2013). Statistical significance was set at *p <* 0.05.

## Results

There were no significant differences in the averaged values of mean power output between the two conditions (488 ± 38 W vs. 486 ± 33 W for normoxic vs. hypoxic recovery, *p* = 0.62).

**FIGURE 2:**
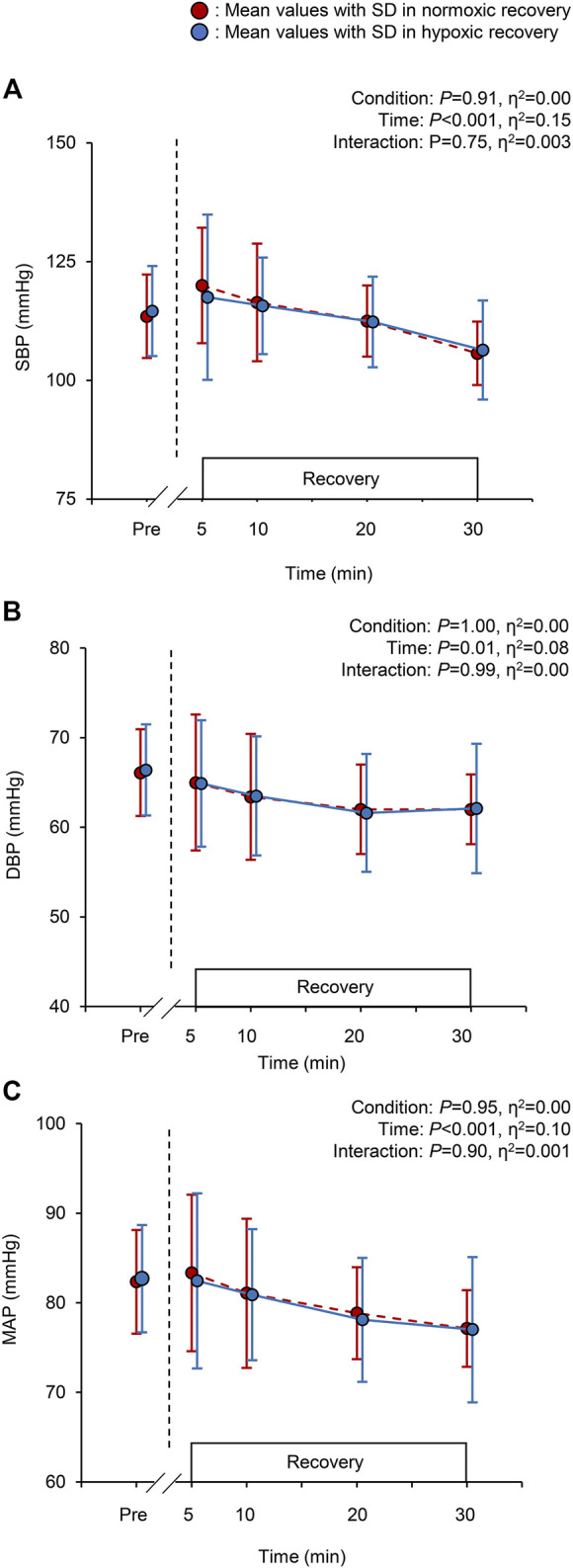
Changes in the systolic blood pressure [SBP; panel **(A)**], diastolic blood pressure [DBP; panel **(B)**], and mean arterial pressure [MAP; panel **(C)**] at baseline (pre) and during the 30-min recovery under normoxia and hypoxia. Values are the mean ± standard deviation (SD). The red and blue circles indicate normoxic and hypoxic recovery conditions, respectively.

The changes in SBP, DBP, and MAP are shown in Figure 2. A significant main effect of time was observed in SBP, DBP, and MAP (all *p* < 0.001, Figures 2A–C), whereas there were no significant condition effects and interactions for these BP variables (all *p* > 0.05, Figures 2A–C). The mean PEH response (differences between values pre-exercise and at 30 min) for SBP, DBP, and MAP in normoxic recovery were −7.8 ± 8.5, −4.1 ± 3.1, and −5.3 ± 4.6 mmHg respectively, and in hypoxic recovery −8.2 ± 6.8, −4.3 ± 4.6, and −5.6  ± 4.8 mmHg, respectively.

**FIGURE 3:**
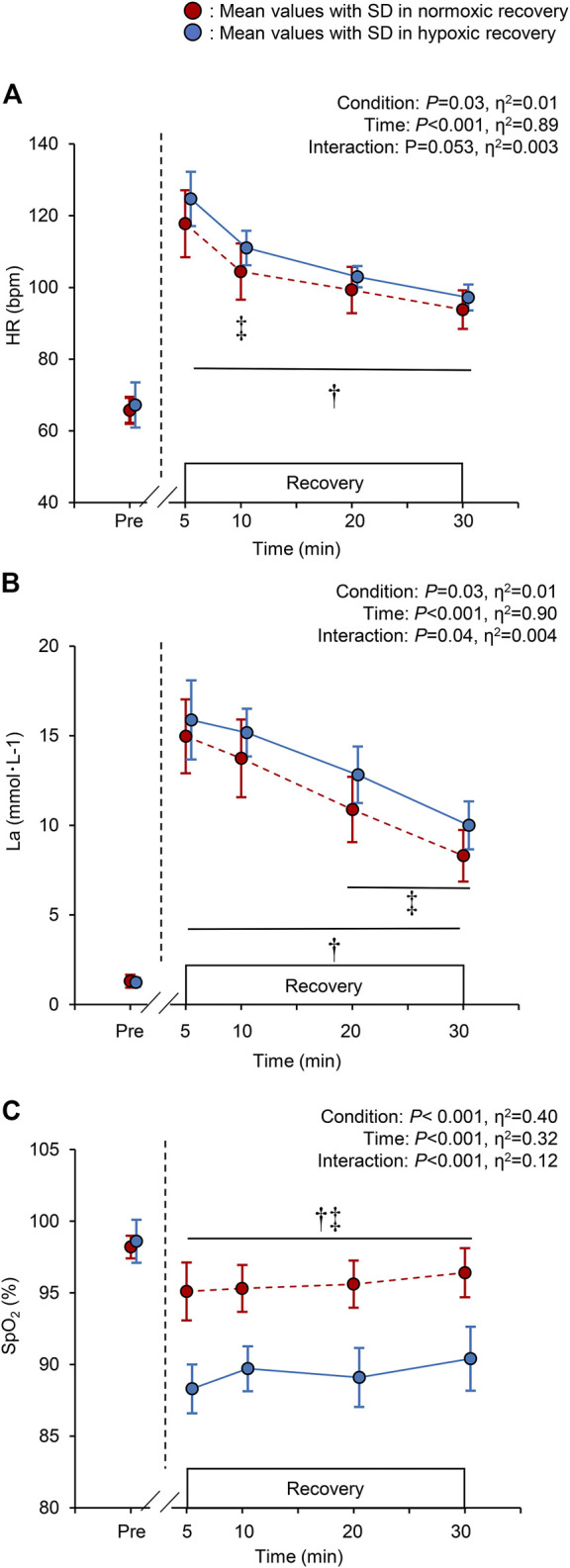
Changes in the heart rate [HR; panel **(A)**], blood lactate concentration [La; panel **(B)**], and peripheral arterial oxygen saturation [SpO_2_; panel **(C)**], at baseline (pre) and during the 30-min recovery under normoxia and hypoxia. All the markers are the same as in Figure 2. †*p* < 0.05 vs. pre-values in both the normoxic and hypoxic recovery conditions, ‡*p* < 0.05 between normoxic and hypoxic recovery conditions at the indicated time point.

There were significant main effects of time and recovery condition on HR, La, and SpO_2_ (all *p* < 0.05. Figures 3A–C). During hypoxic recovery, HR at 10 min was significantly higher than that in normoxic recovery (*p* = 0.03), and showed marginal higher values at 5, 20, and 30 min (all *p* < 0.1 Figure 3A). La at 20 and 30 min during hypoxic recovery was significantly higher than those in normoxic recovery (*p* = 0.02, respectively; Figure 3B). SpO_2_ during hypoxic recovery was significantly lower than normoxic recovery throughout the recovery period (all *p* < 0.05, Figure 3C). Specifically, the mean values of SpO_2_ during recovery were 95.6 ± 1.8% in normoxia and 88.7 ± 1.9% in hypoxia.

**FIGURE 4:**
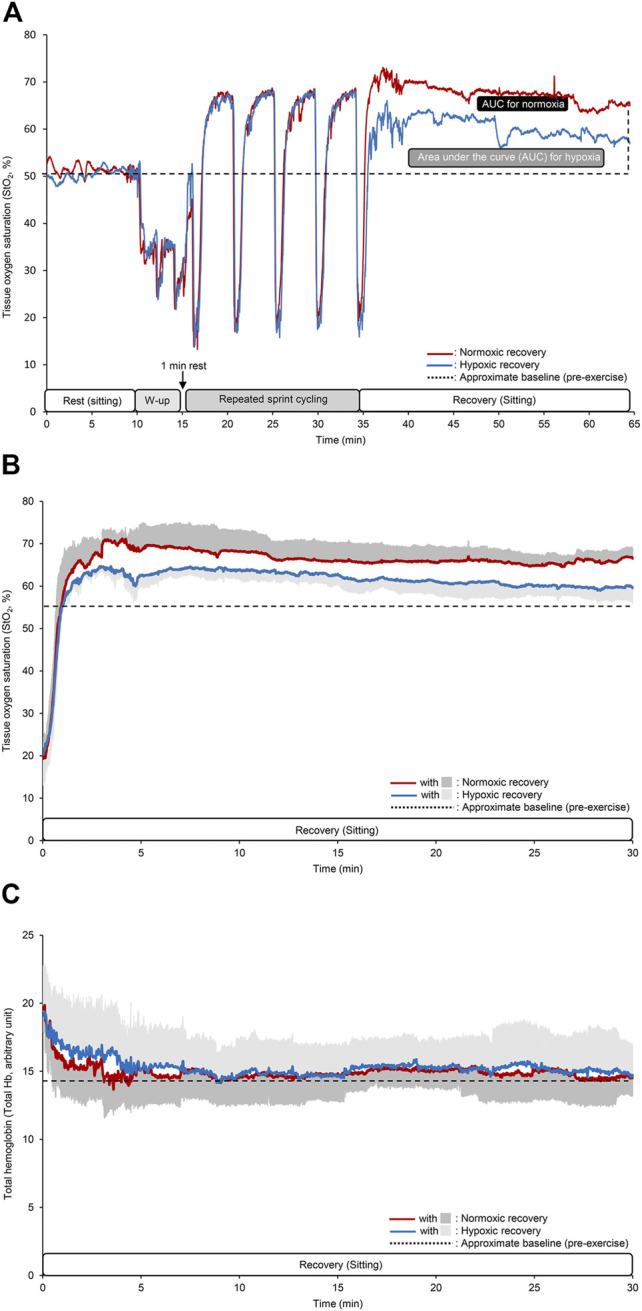
Time course changes in the tissue oxygen saturation (StO_2_) at the vastus lateralis muscle for a typical single subject **(A)**, mean values of StO_2_ and total hemoglobin (Hb) during the recovery period **(B,C)**. The different colored lines indicate the same as that in Figure 2. In the panels **(B,C)**, dark and light gray areas indicate SD for normoxic and hypoxic recovery, respectively, and black dotted lines indicate approximate baseline (pre-exercise) values. Note that SD area are given only upper or lower side.

Time course changes in StO_2_ for a typical single subject and mean values of StO_2_ and total Hb during the recovery period are shown in Figure 4. At rest, during warm-up, and sprint interval leg cycling exercise, the StO_2_ showed similar values between the two conditions. Conversely, the StO_2_ during hypoxic recovery appeared to be markedly lower than that during normoxic recovery. Between the two recovery conditions, baseline (54.9 ± 2.9% vs. 54.7 ± 2.7% for normoxic vs. hypoxic recovery, *p* = 0.74, Cohen’s d = 0.11) and nadir values (20.2 ± 6.2% vs. 21.2 ± 6.9%, *p* = 0.40, Cohen’s d = −0.28) at the five sets of the sprint interval leg cycling exercise were not statistically different. By contrast, peak values (70.8 ± 2.8% vs. 66.8 ± 2.3% for normoxic vs. hypoxic recovery, *p* = 0.02, Cohen’s d = 0.90) and StO_2_ AUC in normoxic recovery (14,500 ± 6,794%･s) were significantly greater than hypoxic recovery (9,535 ± 2,206%･s, *p* = 0.04, Cohen’s d = 0.75) (Figures 4A,B). After exercise, total Hb decreased exponentially within 10 min, and showed similar values as the baseline (pre-exercise) values, irrespective of recovery conditions. There were no significant differences in the mean values of total Hb during the recovery (14.7 ± 1.9 arbitrary unit in normoxia and 15.3 ± 2.3 arbitrary unit in hypoxia, *p* = 0.62, Cohen’s d = 0.16) (Figure 4C).

## Discussion

The present study demonstrated the three findings as follows: 1) MAP significantly decreased over time during the 30-min recovery period after a sprinting exercise in hypoxia, with no difference depending on this recovery is performed in normoxia or hypoxia, 2) HR and La in hypoxic recovery were significantly higher than those in normoxic recovery, and 3) SpO_2_ and StO_2_ AUC during hypoxic recovery were significantly lower than those during normoxic recovery. These results suggest that post-exercise hypotension was not affected by either nomoxic or hypoxic recovery condition despite marked differences in arterial and muscle tissue oxygen saturation, during recovery between the two conditions.

Results showed that compared to pre-exercise (baseline) values, MAP at 30 min during hypoxic recovery decreased by approximately ∼6 mmHg, and by approximately ∼5 mmHg during normoxic recovery, supporting the occurrence of PEH, independently of the conditions. The results coincide with previous studies that found the presence of PEH after hypoxic exercise (Horiuchi et al., 2016; Horiuchi et al., 2018; Kleinnibbelink et al., 2020). Theoretically, a reduction in MAP can be caused by a decrease in HR, stroke volume, or total vascular resistance (TVR). It has been suggested that PEH may be explained by a decrease in TVR, likely due to a combination of central and locally mediated vasodilation, which is not compensated to maintain cardiac output (Halliwill et al., 2014). Since stroke volume has been reported to be unchanged (Talbot et al., 2005) or reduced (Stembridge et al., 2015) in hypoxia, higher HR during hypoxic recovery in the present study may suggest the possibility of maintaining cardiac output. The elevated HR in hypoxic recovery may be explained by hypoxia-induced chemoreceptor stimulation promoting greater sympathetic activation (Bärtsch and Gibbs, 2007; Fisher, 2015) and more commonly, is thought to preserve oxygen delivery. Changes in the TVR is another consideration as it is proven that hypoxia is a powerful vasodilator for peripheral arteries (Dinenno et al., 2003), subsequently leading to a decrease in TVR. Additionally, hypoxic condition has been linked to vasodilation of the splanchnic (Rowell and Blackmon, 1989; Westendorp et al., 1997) and of cutaneous circulation (Simmons et al., 2010; Simmons et al., 2011). As we did not evaluate TVR, we must acknowledge that effects of TVR on PEH is highly speculative; however, the results of the present study might indicate that changes in TVR did not affect PEH in hypoxic recovery condition. Further studies measuring stroke volume, cardiac output, and vascular conductance are required.

Blood lactate concentration as another candidate promoting PEH was higher in hypoxic recovery than in normoxic recovery. An animal experiment found dose-dependent vasodilation by administration of lactate in isolated small arteries (Chen et al., 1996). However, significant differences in the La at 20 and 30 min of normoxic and hypoxic recovery (Figure 3B) in our study did not support the aforementioned hypothesis that vasodilation occurs due to elevated blood lactate concentration.

The StO_2_ AUC during recovery (reactive hyperemia) was markedly greater in normoxic recovery than in hypoxic recovery. In both conditions, the participants performed maximal cycling which resulted in successive intense ischemic episodes in the exercising muscle (i.e., StO_2_ < 20%, Figure 4A). Previous studies have evaluated muscle reoxygenation responses (oxygen delivery to tissues) after exercise (Kemps et al., 2009; Mankowski et al., 2017; Zebrowska et al., 2021), and confirmed the validity and utility of NIRS device when assessing individuals’ recovery kinetics between different oxygen levels (Mankowski et al., 2017). Arterial inflow to thigh muscles may have increased during recovery irrespective of different oxygen recovery conditions, and that normoxic recovery further enhanced these responses compared to hypoxic recovery, probably due to greater arterial O_2_ supply (higher arterial O_2_ content). We found no differences in total Hb responses during recovery between the two conditions. It has been reported that changes in total Hb are associated with changes in blood flow (Alvares et al., 2020) and in vascular conductance (Watanabe et al., 2005). Thus, our results suggest that leg vascular conductance may not vary between normoxic and hypoxic recovery, causing no differences in PEH between the two conditions. Although we can only speculate, regional differential vascular conductance might be associated with the similar PEH finding. It is known that hypoxic conditions promote vasodilation in skeletal muscles during exercise (Dinenno et al., 2003), and we can expect hyperventilation-induced hypocapnia during hypoxic recovery. A previous study demonstrated hypocapnia-induced reductions in cerebral oxygenation and total hemoglobin, suggesting cerebral vasoconstriction (Rupp et al., 2014). Similarly, acute exposure to hypoxia increases pulmonary vasoconstriction (Bärtsch and Gibbs, 2007). In contrast, hypoxia causes cutaneous vasodilation (Simmons et al., 2007) and vasodilation of the splanchnic circulation (Rowell and Blackmon, 1989; Westendorp et al., 1997). Given these possibilities, the mechanisms linking hypoxic conditions and BP regulation are likely complex and multi-factorial, and thus, future studies with measurements of regional vascular conductance may be required.

### Methodological Considerations

First, only young men were enrolled in this study, which makes the results inapplicable to other populations, such as women and elite sport athletes. Second, the present study did not conduct normoxic exercise, followed by normoxic recovery as a control condition. However, our main aim was to compare between normoxic and hypoxic recovery after sprint interval leg cycling exercise in hypoxia. Although future studies are needed, our results may have following future outlooks. Finally, we evaluated only StO_2_ among the indicators of NIRS technique. Venous occlusion technique has been suggested to estimate O_2_ supply and consumption in working muscles during exercise (Homma et al., 1996). Similarly, a recent study demonstrated that combination of arterial and venous occlusion provides valid estimates of blood flow and oxygen consumption at rest and during exercise (Dennis et al., 2021). Although we must acknowledge that these techniques are useful and that we should have performed this technique to obtain more insight interpretations, we considered that further arterial occlusion may not be required after exhaustion. This is because, in the preliminary test, most of the participants complained of intolerable pain and nausea during arterial occlusion after exercise. Thus, we considered a safety for participants preferentially in the present study.

### Perspectives

Sprint interval training in hypoxia has been considered to be a useful strategy to improve exercise both at sea level and high-altitude (Kon et al., 2015). Similarly, it was reported that repeated-sprint training, defined to consist of the repetition of short (<30 s) “all-out” sprints with incomplete recoveries (<60 s) (Girard et al., 2020), in hypoxia significantly improved cardiorespiratory fitness level (Camacho-Cardenosa et al., 2020). Additionally, even though under normoxic environments, a recent review summarized that sprint interval training improves glucose metabolism (Jiménez-Maldonado et al., 2020). Although these previous findings are beyond of the present study, studies including various combination of hypoxic (normoxic) exercise and recovery conditions may be required. As the present study is the first study to compare the effects of a normoxic versus hypoxic recovery on post-exercise hemodynamics, the present results may be informative for populations who perform an exercise at high-altitude, particularly from the viewpoint of safe implementation for conducting sprint interval training in hypoxia. For example, while the average magnitude of PEH in the present study was approximately 6 mmHg, regardless of different recovery conditions, previous studies reported PEH magnitudes of approximately 3 mmHg after 45 min of a high-intensity running exercise (85% of maximal HR) (Kleinnibbelink et al., 2020), approximately 6 mmHg after a 2-h leg cycling exercise (50% of peak oxygen uptake) (Horiuchi et al., 2016), and more than 10 mmHg after a resistance exercise (bilateral leg squat) (Horiuchi et al., 2018). These different magnitudes of PEH may be influenced by different study settings. Since these previous studies (Horiuchi et al., 2016; Horiuchi et al., 2018; Kleinnibbelink et al., 2020) and our study did not evaluate symptoms leading to presyncope, it is still uncertain whether sprint interval exercise in hypoxia increases the risk of presyncope. Nevertheless, the current findings suggest that the risks of presyncope may be similar with either normoxic or hypoxic recovery. Moreover, a recent study demonstrated that an acute exercise-induced reduction in BP was related to a greater chronic reduction in the BP-lowering effect of exercise training (Kleinnibbelink et al., 2020). Therefore, the greater magnitude of PEH in the present study, compared with the previous study (Kleinnibbelink et al., 2020)**,** might indicate that regular sprint interval training in hypoxia could be a future strategy for antihypertensive therapy.

## Conclusion

Sprint interval leg cycling exercise in hypoxia caused significant reductions in BP variables, regardless of hypoxic and normoxic recovery. SpO_2_ and StO_2_ AUC were also lower than that with normoxic recovery. These results suggest that PEH after sprint interval leg cycling exercise in hypoxia was not affected by either normoxic or hypoxic recovery despite marked differences in SpO_2_ and StO_2_ between the two recovery conditions.

## Data Availability

The original contributions presented in the study are included in the article/Supplementary Materials, further inquiries can be directed to the corresponding author.
